# An E2–F12 complex is required for intracellular enveloped virus morphogenesis during vaccinia infection

**DOI:** 10.1111/j.1462-5822.2009.01296.x

**Published:** 2009-02-27

**Authors:** Mark P Dodding, Timothy P Newsome, Lucy M Collinson, Ceri Edwards, Michael Way

**Affiliations:** 1 Cell Motility Laboratory, Cancer Research UK, London Research Institute44 Lincoln's Inn Fields, London, WC2A 3PX, UK; 2 Electron Microscopy Unit, Cancer Research UK, London Research Institute44 Lincoln's Inn Fields, London, WC2A 3PX, UK

## Abstract

The vaccinia virus protein, F12, has been suggested to play an important role in microtubule-based transport of intracellular enveloped virus (IEV). We found that GFP-F12 is recruited to IEV moving on microtubules but is released from virus particles when they switch to actin-based motility. In the absence of F12, although the majority of IEV remain close to their peri-nuclear site of assembly, a small number of IEV still move with linear trajectories at speeds of 0.85 μm s^−1^, consistent with microtubule transport. Using a recombinant virus expressing GST-F12, we found that the viral protein E2 interacts directly with F12. In infected cells, GFP-E2 is observed on moving IEV as well as in the Golgi region, but is not associated with actin tails. In the absence of E2L, IEV accumulate in the peri-nuclear region and F12 is not recruited. Conversely, GFP-E2 is not observed on IEV in the absence of F12. Ultra-structural analysis of ΔE2L- and ΔF12L-infected cells reveals that loss of either protein results in defects in membrane wrapping during IEV formation. We suggest that E2 and F12 function as a complex that is necessary for IEV morphogenesis prior to their microtubule-based transport towards the plasma membrane.

## Introduction

Vaccinia virus is a large double-stranded DNA virus that undergoes a complex replication cycle in the cytoplasm of the host cell (Schramm and Locker, 2005; Condit *et al*., 2006; Moss, 2007; Roberts and Smith, 2008). Viral replication, which occurs in specialized virus factories localized near the microtubule-organizing centre, results in the formation of the intracellular mature virus (IMV). Infectious IMV are released when the infected cell undergoes lysis but are also capable of being transported out of the virus factory in a microtubule-dependent fashion (Sanderson *et al*., 2000; Ward, 2005). A proportion of these IMV can subsequently be wrapped by membrane cisternae derived either from the trans-Golgi network or endosomes containing a subset of integral viral membrane proteins, to become intracellular enveloped virus (IEV) particles (Smith *et al*., 2002; Roberts and Smith, 2008). Once formed, IEV are transported from their peri-nuclear site of formation to the cell periphery on microtubules by kinesin-1 (Geada *et al*., 2001; Hollinshead *et al*., 2001; Rietdorf *et al*., 2001; Ward and Moss, 2001a;b).

Upon reaching the cell periphery, IEV undergo actin-dependent movements in the cell cortex until they fuse with the plasma membrane (Arakawa *et al*., 2007b). After fusion, a proportion of extracellular virus particles known as a cell-associated enveloped virus (CEV) remain attached to the plasma membrane and induce an outside-in signalling cascade that locally activates Src and Abl family kinases (Frischknecht *et al*., 1999; Reeves *et al*., 2005). This activation results in phosphorylation of tyrosines 112 and 132 of A36, an integral IEV membrane protein that becomes localized beneath CEV following fusion of IEV with the plasma membrane (Frischknecht *et al*., 1999; van Eijl *et al*., 2000; Newsome *et al*., 2004; 2006). Phosphorylation of tyrosine 112 results in the recruitment of a complex consisting of Nck, WIP and N-WASP, beneath the CEV (Frischknecht *et al*., 1999; Moreau *et al*., 2000; Snapper *et al*., 2001; Zettl and Way, 2002; Weisswange *et al*., 2009). Phosphorylation of tyrosine 132 of A36 and the presence of the polyproline-rich region of N-WASP results in the recruitment of Grb2, which in turn stabilizes a complex of Nck : WIP : N-WASP beneath the virus (Scaplehorn *et al*., 2002; Weisswange *et al*., 2009). Ultimately, the presence of N-WASP locally stimulates the actin-nucleating activity of the Arp2/3 complex beneath the CEV, resulting in the formation of an actin tail, which helps propel the virus into neighbouring cells (Cudmore *et al*., 1995; 1996).

The efficient cell-to-cell spread of vaccinia virus is dependent on microtubule-mediated transport of IEV particles as well as the formation of actin tails at the plasma membrane beneath CEV (Geada *et al*., 2001; Hollinshead *et al*., 2001; Ward and Moss, 2001b; a; Roberts and Smith, 2008). The integral IEV membrane protein, A36, which is essential for actin tail formation (Sanderson *et al*., 1998; Wolffe *et al*., 1998), also plays an important role in microtubule-based transport of IEV particles (Rietdorf *et al*., 2001; Ward and Moss, 2001b). Deletion of the A36R gene from the vaccinia genome leads to the loss of kinesin-1 recruitment to IEV particles, resulting in loss of their efficient transport to the cell periphery (Rietdorf *et al*., 2001; Ward and Moss, 2001a; b). It is thought that A36 is directly responsible for kinesin-1 recruitment, as it is able to interact with the light chain (KLC) of the microtubule motor (Ward and Moss, 2004). The only other viral protein that has been implicated in microtubule-mediated transport of IEV particles is F12 (van Eijl *et al*., 2002). F12, which is conserved in all chordopoxviruses and lacks any transmembrane domains (Zhang *et al*., 2000), is recruited to IEV associated with microtubules but is absent from the tips of actin tails (van Eijl *et al*., 2002). Moreover, deletion of the F12L gene from the viral genome results in a small plaque phenotype, the accumulation of IEV particles at their peri-nuclear site of formation and a severe reduction of actin tail formation (van Eijl *et al*., 2002). These findings have led to the suggestion that the protein may play an important role in microtubule-based motility of IEV (van Eijl *et al*., 2002).

In the current study, we set out to clarify the role of F12 during IEV transport using live cell imaging. Unexpectedly, we found that F12 is not essential for the microtubule-based transport of IEV moving to the cell periphery. F12 does, however, interact directly with the vaccinia E2 protein, which is recruited to IEV in an F12-dependent fashion. Moreover, loss of either protein results in IEV membrane wrapping defects, suggesting that the primary role of the E2–F12 complex is in the morphogenesis rather than the microtubule-based transport of IEV.

## Results

### GFP-F12 associates with moving IEV

**Fig. 1:**
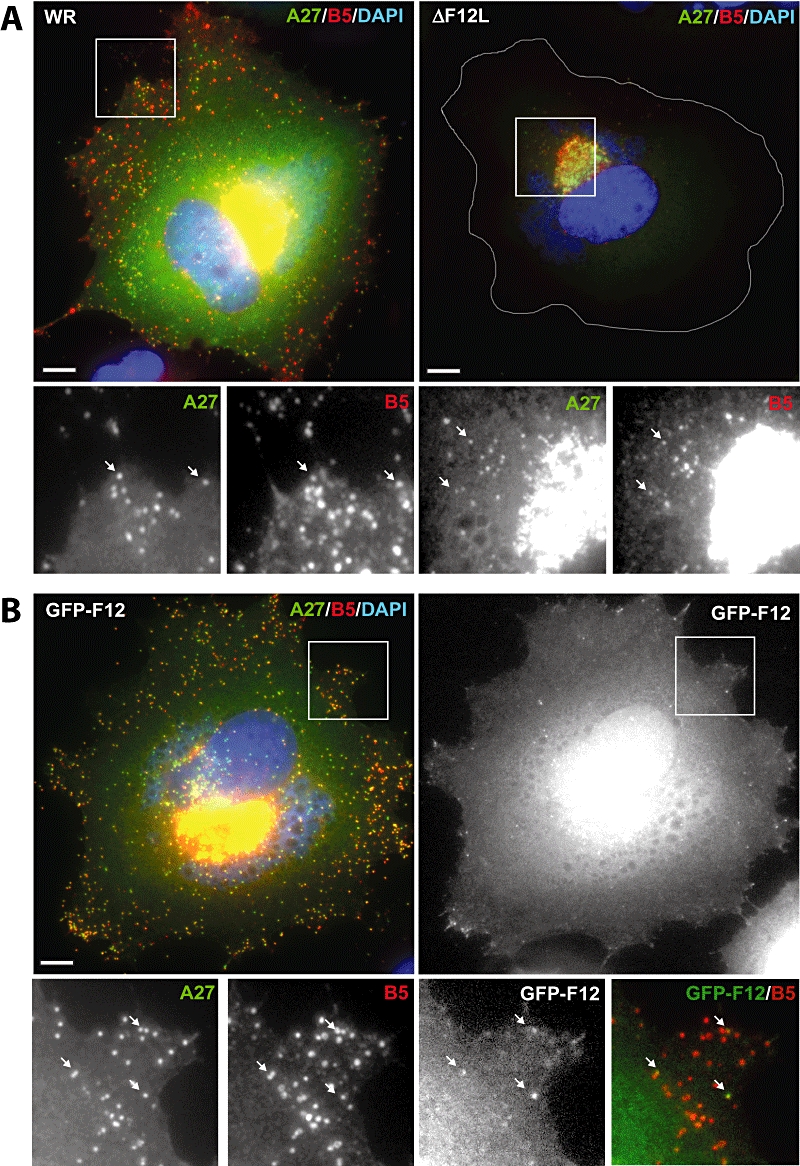
GFP-F12 is associated with IEV. Immunofluorescence images of WR- and ΔF12L- (A) and WR-GFP-F12- (B) infected HeLa cells at 8 h post infection labelled with antibodies against A27 (green) and B5 (red) as well as DAPI (blue) to visualize DNA. The GFP-F12 signal in (B) is shown in black and white. Higher-magnification images correspond to the boxes in the main panels and arrows indicate A27/B5- and A27/B5/GFP-F12-positive IEV in (A) and (B) respectively. The scale bar is 10 μm.

**Fig. 2:**
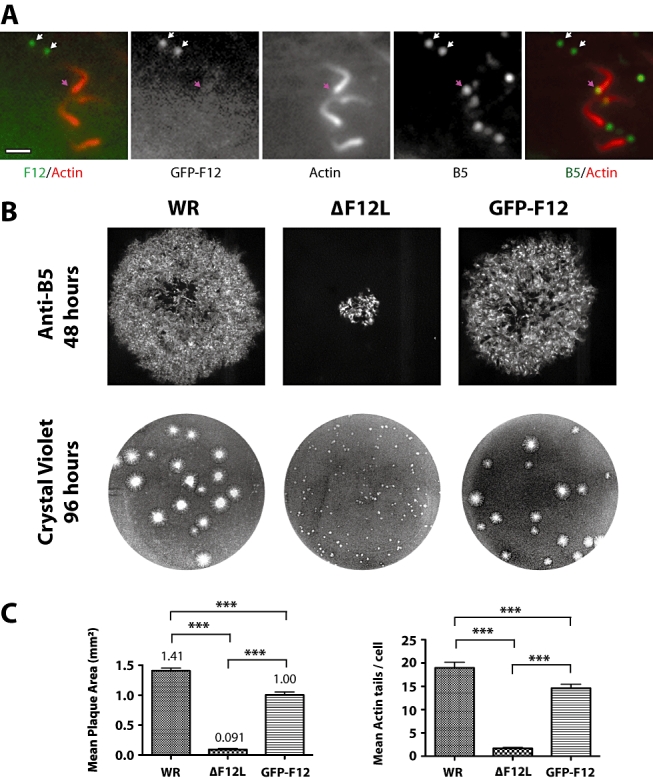
GFP-F12 rescues actin tail formation and cell-to-cell spread of ΔF12L virus. A. Immunofluorescence images of WR-GFP-F12-infected HeLa cells reveals that GFP-F12 is associated with B5-positive IEV particles (white arrows) but is absent from the tips of actin tails (pink arrow). Scale bar = 2 μm. B. Representative images of plaques formed by WR, ΔF12L and WR-GFP-F12 in confluent BS- C-1 monolayers at 48 (anti-B5) and 96 h (crystal violet) post infection. C. Quantitative analysis of plaque size at 48 h post infection and the number of actin tails per cell in WR, ΔF12L and WR-GFP-F12 8 h post infection. Error bars represent the SEM and were derived from measuring the area of 20 plaques or counting 150 infected HeLa cells. ***P < 0.001.

To obtain further insights into the role of F12 during viral egress, we created a recombinant virus expressing GFP-F12 using homologous recombination by rescuing the ΔF12L virus (Zhang *et al*., 2000). We found that GFP-F12 was recruited to IEV particles positive for A27 and B5 or DAPI and F13, as well as a peri-nuclear B5-positive membrane compartment corresponding to the trans-Golgi network (Fig. 1B; Fig. S1). GFP-F12 was also able to rescue the egress defect observed in ΔF12L-infected cells (Fig. 1A and B). GFP-F12 was, however, absent from the tips of actin tails formed beneath CEV (Fig. 2A). Plaque assays confirmed that GFP-F12 was largely able to rescue the defect in the cell-to-cell spread of the ΔF12L virus (Fig. 2B and C). Quantification of actin tail formation also revealed a similar degree of rescue (Fig. 2C).

**Fig. 3:**
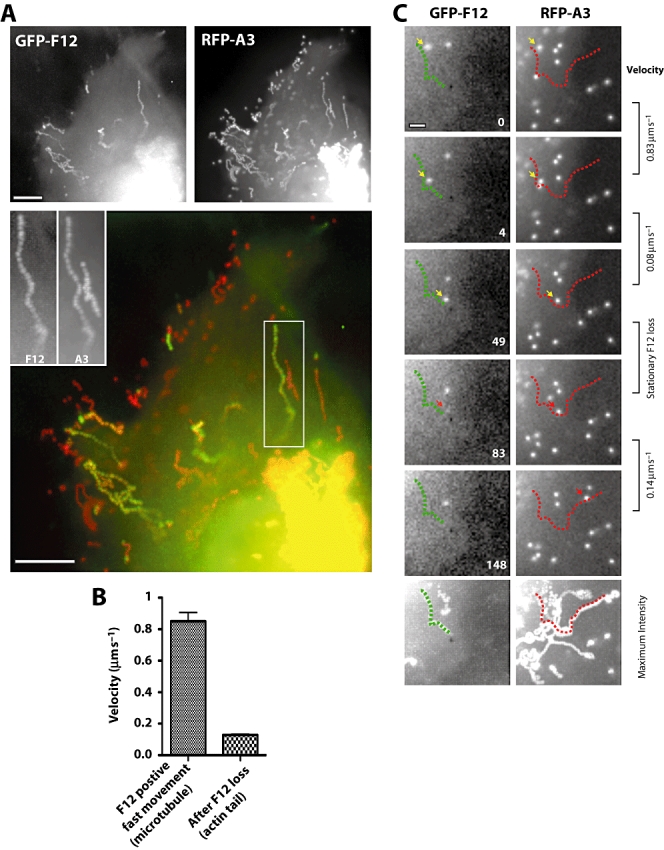
GFP-F12 is associated with rapidly moving IEV. A. Images of the maximum intensity projection of GFP-F12 and RFP-A3 associated with moving IEV over a period of 52 s in a WR-GFP-F12-infected cell (Movie S1). The GFP-F12 and RFP-A3 signals were acquired simultaneously using a fluorescent beam splitter and the scale bar is 10 μm. B. The graph shows the average speed of directional linear IEV movements (microtubule-based) prior to disappearance of F12 and the subsequent slower processive movements (actin-based). Error bars indicate SEM and *n* = 20. C. Movie stills taken from Movie S2 showing that GFP-F12 is lost from IEV when they become stationary in the cell periphery, as they switch to slower more processive actin-based motility after fusion with the plasma membrane (Movie S2). Time in seconds and the average velocity between the indicated frames are shown. Scale bar = 2 μm.

To characterize the spatial and temporal recruitment of GFP-F12 to IEV, we performed live cell imaging (Fig. 3; Movie S1). To ensure the correct identification of authentic IEV particles from GFP-F12-positive endosomes (van Eijl *et al*., 2002), we also expressed an RFP-tagged A3 viral core marker (Weisswange *et al*., 2009) during infection. Infected cells were imaged at 8 h post infection and the signals from GFP-F12 and RFP-A3 were acquired simultaneously (Fig. 3A). IEV particles positive for both GFP-F12 and RFP-A3 were observed to move rapidly in a linear fashion towards the cell periphery with an average speed of 0.85 ± 0.06 μm s^−1^ (Fig. 3A and B; Movie S1). This speed is consistent with values previously obtained for microtubule-based transport of IEV particles (Geada *et al*., 2001; Hollinshead *et al*., 2001; Rietdorf *et al*., 2001; Ward and Moss, 2001a; b). Interestingly, when many of the IEV reach the cell periphery we observed that the GFP-F12 signal vanishes over a period of less than 30 s prior to RFP-A3-positive virus particles switching to a slower, more processive form of movement (Fig. 3C; Movie S2). The average speed of this slower movement is 0.13 ± 0.005 μm s^−1^, which is consistent with actin tail-based motility (Cudmore *et al*., 1995; Rietdorf *et al*., 2001; Arakawa *et al*., 2007b; Weisswange *et al*., 2009). These live cell observations suggest that F12 is lost from IEV particles as they fuse with the plasma membrane, which is consistent with the lack of GFP-F12 association beneath CEV (Fig. 2A).

### F12 is not required for microtubule-based IEV movement

**Fig. 4:**
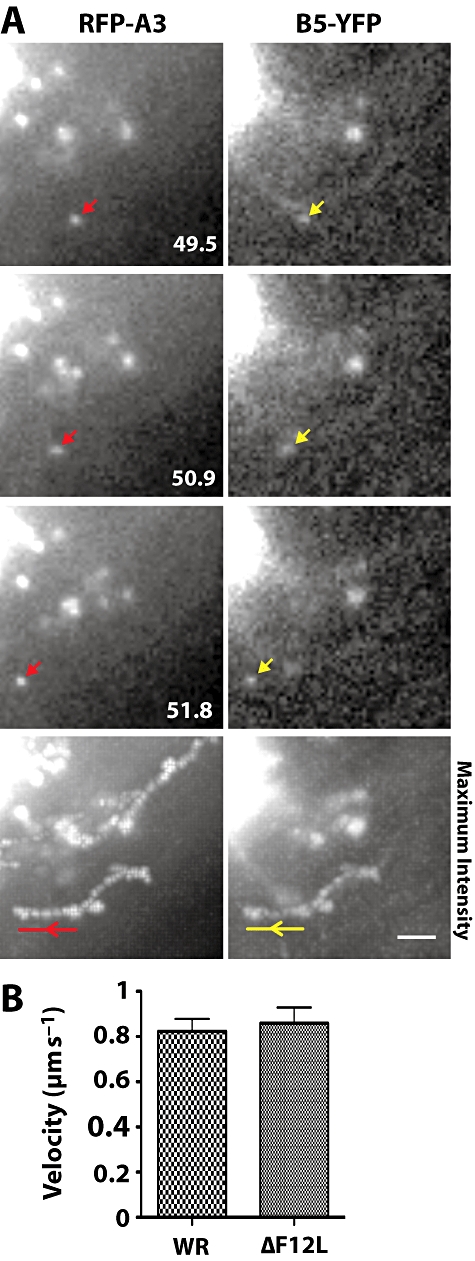
F12 is not required for microtubule-mediated transport of IEV. A. Movie stills taken from Movie S3 at the indicated times and the maximum intensity projection of B5-YFP and RFP-A3 associated with a moving IEV (arrows) over a period of 64 s in a WR-ΔF12L/B5-YFP/RFP-A3-infected cell. The B5-YFP and RFP-A3 signals were acquired simultaneously using a fluorescent beam splitter and the scale bar is 2 μm. B. The graph shows the average velocity of B5-YFP- and RFP-A3-positive IEV particles moving in linear trajectories in WR-B5-YFP/RFP-A3- and WR-ΔF12L/B5-YFP/RFP-A3-infected cells. Error bars show SEM and *n* = 20.

Our observations as well as those of van Eijl *et al*. (2002) clearly demonstrate that F12 is associated with IEV moving on microtubules. Although there is a correlation between F12 recruitment and the microtubule-based transport of IEV, a direct role for F12 in this process still remains to be established. To examine if F12 is required for microtubule-mediated IEV transport, we generated a recombinant virus expressing B5-YFP in the ΔF12L viral background. B5-YFP represents a very good marker to identify IEV particles (Ward and Moss, 2001a). However, B5 is also recruited to numerous endosomes that are easily confused for virus particles. To ensure an unambiguous identification of IEV, we also ectopically expressed RFP-A3 to provide a marker of the virus core in ΔF12L/B5-YFP-infected cells. Cells infected with Western Reserve (WR)/B5-YFP or ΔF12L/B5-YFP viruses and expressing RFP-A3 were imaged 6–8 h post infection. The signals from B5-YFP and RFP-A3 were acquired simultaneously to ensure the correct identification of rapidly moving IEV from B5-YFP-positive endosomes (Fig. 4A; Movie S3). In the absence of F12 the majority of B5-YFP- and RFP-A3-positive virus particles remained close to the nucleus. However, in every infected cell we still observed a small number of IEV particles that emerged from the peri-nuclear region in the absence of F12. These B5-YFP- and RFP-A3-positive IEV moved at a similar velocity to those formed in its presence (0.82 ± 0.05 compared with 0.86 ± 0.07 μm s^−1^) (Fig. 4B; Movie S3). This movement indicates that F12 is not absolutely required for microtubule-based transport of IEV particles.

### F12 interacts directly with E2

**Fig. 5:**
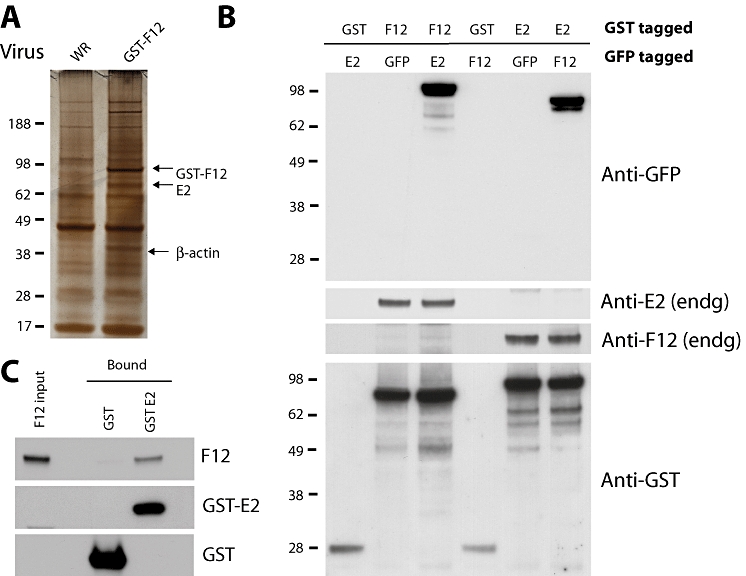
F12 interacts directly with E2. A. A silver stained gel showing that E2, which was identified by mass spectrometry, copurifies with GST-F12 on glutathione beads from cells infected with WR-GST-F12 but not WR. B. Immunoblot analysis with the indicated antibodies of glutathione sepharose pull-downs reveals that GST-tagged E2 and F12 copurify with GFP-tagged as well as the endogenous F12 and E2 respectively. C. Immunoblot analysis of glutathione sepharose pull-downs reveals that GST-E2 but not GST produced in *E. coli* retains His-tagged F12 from an *E. coli* extract.

The molecular basis of F12 recruitment to IEV particles remains unknown. To facilitate identification of potential F12 binding partners we constructed a recombinant virus expressing GST-F12 by rescuing the ΔF12L virus. Glutathione resin pull-down assays on extracts from cells infected with the GST-F12 virus reveals the presence of two proteins, which were absent in the WR control sample (Fig. 5A). Mass spectrometry identified these two proteins as β-actin and the viral protein E2. The band corresponding to β-actin could not be consistently reproduced in repeat experiments and so was not pursued further. To confirm the interaction between F12 and E2, we performed pull-down assays on infected cell extracts expressing GFP- and GST-tagged versions of the two proteins. Pull-down assays using glutathione resin demonstrated that GFP-E2 and GFP-F12 readily copurify with GST-F12 and GST-E2 respectively (Fig. 5B). Using F12- and E2-specific antibodies, we also found that endogenous E2 and F12 copurified with GST-F12 and GST-E2 respectively (Fig. 5B). Although F12 associates with IEV moving on microtubules, we were unable to detect kinesin-1 copurifying with GST-tagged F12 or E2 (data not shown). Pull-down assays from infected cell extracts are indicative, but do not demonstrate that a direct interaction is occurring. We therefore performed pull-down assays using GST-E2 and His-F12 produced in bacteria to investigate whether the two proteins interact directly with each other. Both proteins were soluble in *Escherichia coli* and expressed at the correct predicted size. Moreover, we found that GST-E2 but not GST was able to bind directly to His-tagged F12 (Fig. 5C).

### E2 is associated with moving IEV

**Fig. 6:**
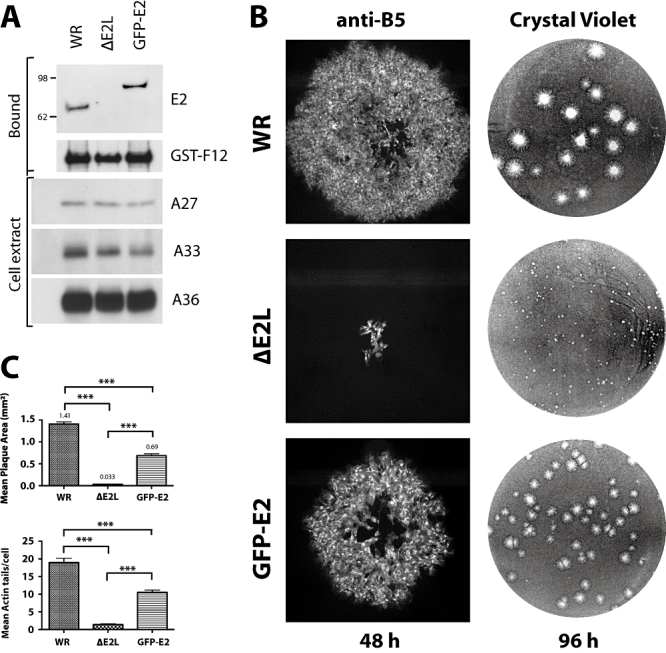
Loss of E2 leads to reduced actin tail formation and cell-to-cell spread. A. Immunoblot analysis of E2 expression in WR-, WR-ΔE2L- or WR-GFP-E2-infected HeLa cells at 10 h post infection. The E2 signal in the infected cell extracts was enriched using a GST-F12 pull-down as the protein is expressed at very low levels. The expression levels of A27, A33, A36 in the infected cell extracts used for the GST-F12 pull-down are provided as loading controls. B. Representative images of plaques formed by WR, ΔE2L and WR-GFP-E2 in confluent BS- C-1 monolayers at 48 (anti-B5) and 96 h (crystal violet) post infection. C. Quantitative analysis of plaque size at 48 h post infection and the number of actin tails per cell in WR, ΔE2L and WR-GFP-E2 at 8 h post infection. Error bars representing the SEM were derived from measuring the area of 20 plaques or counting 150 infected HeLa cells. ***P < 0.001.

**Fig. 7:**
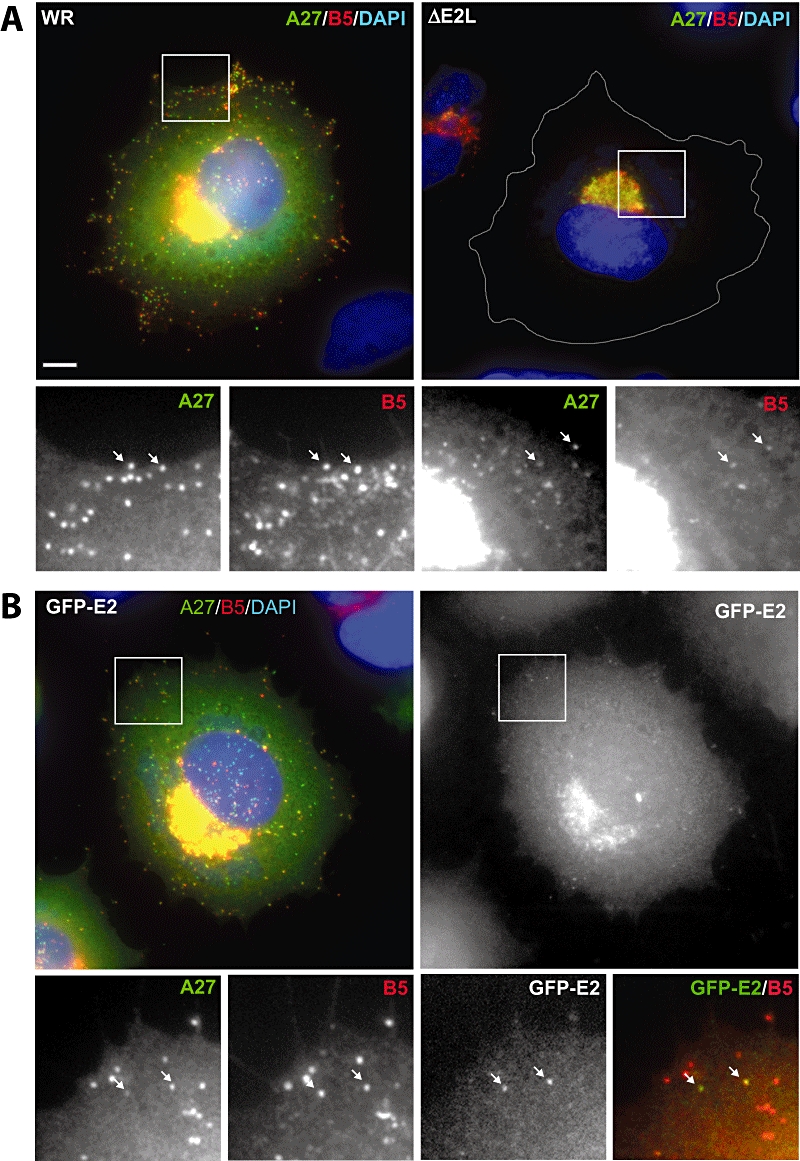
E2 is required for the egress of IEV to the cell periphery. Immunofluorescence images of WR- and ΔE2L- (A) and WR-GFP-E2- (B) infected HeLa cells at 8 h post infection labelled with antibodies against A27 (green) and B5 (red) as well as DAPI (blue) to visualize DNA. The GFP-E2 signal is shown in black and white in (B). Higher-magnification images corresponding to the boxes in the main panels and white arrows indicate A27/B5- and A27/B5/GFP-E2-positive IEV in (A) and (B) respectively. The scale bar is 10 μm.

**Fig. 8:**
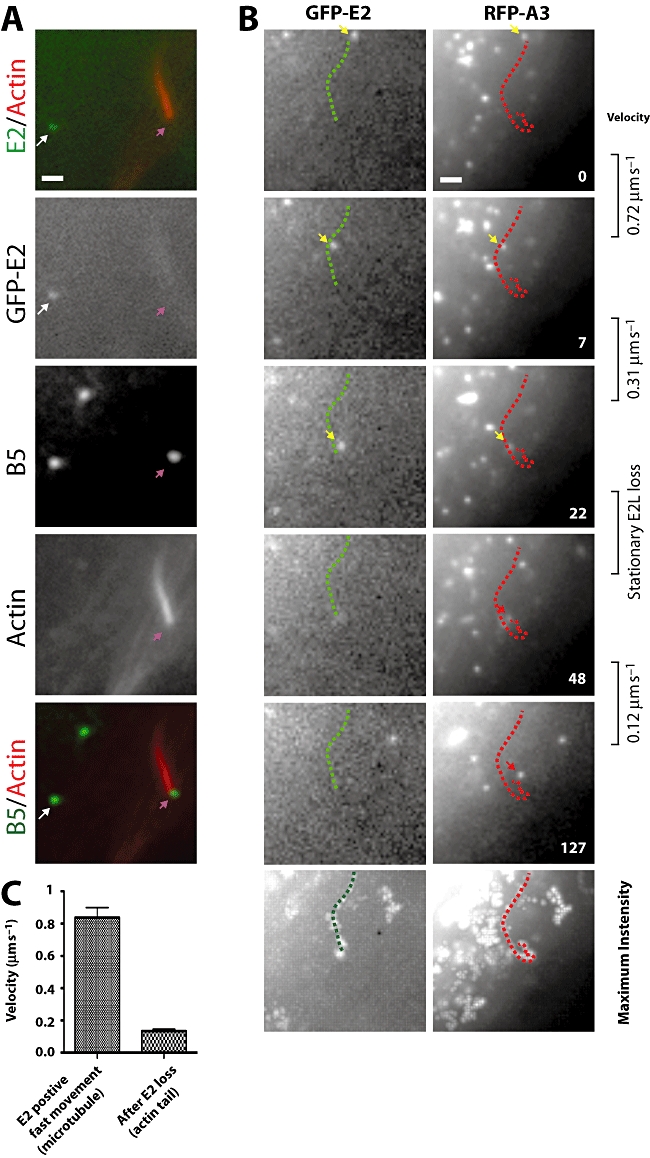
Movement of GFP-E2-positive virus particles. A. Immunofluorescence images of WR-GFP-E2-infected HeLa cells reveals that GFP-E2 is associated with B5-positive IEV particles (white arrow) but is absent from the tips of actin tails (pink arrow). Scale bar = 2 μm. B. Movie stills taken from Movie S4 showing that GFP-E2 is lost from IEV when they become stationary in the cell periphery, as they switch to slower more processive actin-based motility after fusion with the plasma membrane (Movie S4). Time in seconds and the average velocity between the indicated frames are shown. Scale bar = 2 μm. C. The graph shows the average speed of directional linear IEV movements (microtubule-based) prior to disappearance of E2 and the subsequent slower processive movements (actin-based). Error bars show SEM and *n* = 20.

To examine the role of E2 during vaccinia infection, we generated a recombinant virus in which we deleted the E2L gene by replacing it with a gpt/Cherry cassette under the control of synthetic early/late promoters (Fig. 6A). The ΔE2L virus has a very small plaque phenotype and makes very few actin tails (Fig. 6B and C). These properties, which are reminiscent of the ΔF12L virus, are consistent with possible defects in IEV egress to the cell periphery. Immunofluorescence analysis of ΔE2L-infected cells confirmed that IEV particles remain largely in the peri-nuclear region in the absence of E2 (Fig. 7A). To help understand the role of E2 in the movement of IEV to the cell periphery, we created a recombinant virus expressing GFP-E2 by homologous recombination (Fig. 6A). Plaque assays demonstrated that GFP-E2 was able to partially rescue the cell-to-cell spread and actin tail defects of the ΔE2L virus, although not to the same extent as GFP-F12 (Fig. 6B and C). Immunofluorescence analysis reveals that GFP-E2 colocalizes with B5 on Golgi apparatus and IEV particles but absent from IMV (Fig. 7B). As observed with F12, GFP-E2 was not associated with the tips of actin tails induced by CEV (Fig. 8A). Live cell imaging at 8 h post infection reveals that GFP-E2 is associated with RFP-A3-positive IEV particles moving with an average speed of 0.84 ± 0.06 μm s^−1^ (Fig. 8B and C). This value indicates that E2 is also associated with IEV moving on microtubules. GFP-E2 was also seen to dissociate from RFP-A3-positive virus particles when they switch to the slower actin-based motility in the cell periphery (Fig. 8B; Movie S4).

### E2 and F12 are recruited to IEV as a complex

**Fig. 9:**
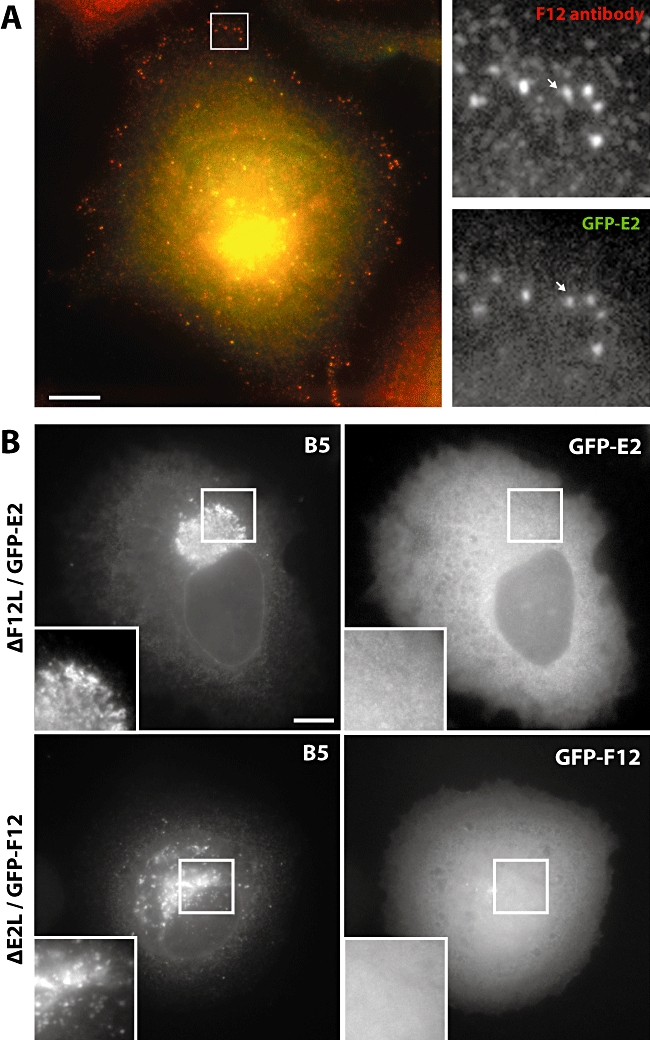
Association of GFP-E2 with F12. A. Immunofluorescence images demonstrating that GFP-E2 colocalizes with F12 in HeLa cells infected with WR-GFP-E2 at 8 h post infection. B. Immunofluorescence images of cells infected with ΔF12L or ΔE2L viruses reveals that the presence of both E2 and F12 is required for their recruitment to IEV and the Golgi (B5).

The phenotype of the ΔE2L and ΔF12L viruses and localization of the two proteins are essentially identical. Moreover, immunofluorescence analysis of GFP-E2-infected cells using an F12 antibody confirmed that both proteins colocalized with each other on IEV particles, consistent with their ability to interact with each other (Fig. 9A). Given these observations, we tested whether E2 and F12 function as a complex or if one protein is responsible for mediating recruitment of the other. We found that neither GFP-tagged E2 nor F12 was recruited to IEV particles or the Golgi region in ΔF12L- or ΔE2L-infected cells respectively (Fig. 9B). This suggests that both proteins are recruited and function as a complex.

**Fig. 10:**
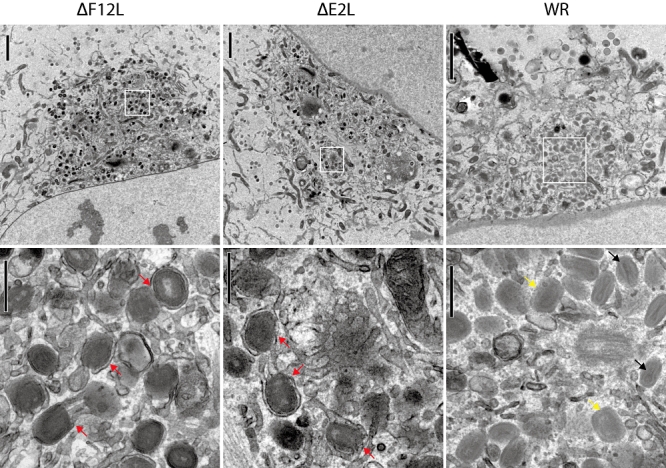
E2 and F12 are required for IEV morphogenesis. Electron micrographs showing the peri-nuclear wrapping compartment in WR-, ΔE2L- and ΔF12L-infected HeLa cells at 8 h post infection. In WR-infected cells IMV (black arrows) and wrapped brick-shaped IEV (yellow arrows) are readily observed. In contrast, in ΔE2L- and ΔF12L-infected cells, IEV are partially wrapped (red arrows) and embedded in a dense tubular network of membranes. Scale bars = 2 μm top panels and 0.5 μm bottom panels.

Recent observations have shown that loss of E2 leads to reduction in IEV formation because of defects in IMV membrane wrapping (Domi *et al*., 2008). In contrast, previous observations indicate that the loss of F12 only affected IEV egress and not their formation (van Eijl *et al*., 2002). Based on our findings that both proteins appear to function as a complex, we decided to re-examine the ultra-structure of cells infected with the ΔF12L virus in the electron microscope (Fig. 10; Figs S2 and S3). In HeLa cells infected with WR for 8 h we were able to detect numerous IEV. In contrast, in both ΔE2L- and ΔF12L-infected cells we observed very few intact IEV and a large number of partially or aberrantly wrapped IMV. It was also noticeable that in contrast to WR-infected cells, the partially wrapped IMV were embedded in extensive tubulated membrane cisternae. Our observations, which are consistent with those of Domi *et al*. (2008), suggest that the primary function of the E2–F12 complex is during IEV formation and not transport to the cell periphery.

## Discussion

During vaccinia virus infection, the efficiency of the cell-to-cell spread of the virus in cell culture is enhanced by the formation of IEV, their transport to the cell periphery and the subsequent stimulation of actin polymerization underneath the CEV (Roberts and Smith, 2008). In the absence of an active transport mechanism, the movement of IEV from their peri-nuclear site of assembly to the plasma membrane would be an inefficient process given their size and the viscosity of the cytoplasm (Sodeik, 2000). However, IEV particles are able to recruit kinesin-1 and undergo plus end-directed microtubule movements to the cell periphery (Geada *et al*., 2001; Hollinshead *et al*., 2001; Rietdorf *et al*., 2001; Ward and Moss, 2001a;b). Currently, only two viral proteins, A36 and F12, have been suggested to play a direct role in microtubule-mediated movement of IEV (Rietdorf *et al*., 2001; van Eijl *et al*., 2002). Loss of either A36 or F12 results in the accumulation of IEV in a peri-nuclear compartment near their site of assembly. A36, an integral IEV membrane protein (Smith *et al*., 2002), appears to be responsible for recruiting the microtubule motor as it can interact directly with the kinesin-1 light chain (Ward and Moss, 2004). In contrast, the role of F12 in egress of IEV towards the plasma membrane remains obscure. In this paper we set out to obtain additional insights into the role of F12 during IEV transport by imaging live cells infected with a recombinant virus expressing GFP-F12 under its endogenous promoter, as all previous F12 studies have used fixed samples.

Our observations in both fixed and live WR-GFP-F12-infected cells have confirmed previous observations that F12 is associated with IEV particles but is absent from IMV (van Eijl *et al*., 2002). In contrast to these early studies, we found that GFP-F12 also associates with a B5-positive peri-nuclear membrane compartment (Golgi region), in addition to moving IEV. This additional localization may reflect differences in using GFP as a tag as compared with an HA antibody whose labelling is dependent on epitope tag accessibility and the fixation conditions. Consistent with this suggestion we observed that GFP-F12 had a similar localization as the endogenous F12 detected with an antibody against F12 (Figs 1B and 9B). As observed previously, we found that F12 is not present beneath CEV inducing actin tails. Moreover, in live cells we also observed that GFP-F12 is released from IEV particles in the cell periphery when they switch to actin-based motility after they fuse with the plasma membrane. The association of GFP-F12 with IEV during their microtubule transport phase is consistent with the suggestion of van Eijl *et al*. (2002) that the protein plays an important role in microtubule-based motility of IEV. This hypothesis is further supported by the observation that in the absence of F12, the vast majority of IEV accumulate in the peri-nuclear region of ΔF12L-infected cells. However, using live cell imaging we have now found that in the absence of F12 a small number of IEV still move towards the cell periphery at 0.85 ± 0.02 μm s^−1^. The behaviour and speed of these viral movements are consistent with previous observations of microtubule-based motility of IEV (Geada *et al*., 2001; Hollinshead *et al*., 2001; Rietdorf *et al*., 2001; Ward and Moss, 2001a;b). It therefore appears that F12 is not essential for transport of IEV on microtubules. This immediately raises the question: what is the role of F12 during the vaccinia virus life cycle?

Our demonstration that F12 interacts directly with E2 now provides new insights into its possible role during the virus life cycle. We found that E2, whose localization was previously undefined, was recruited to IEV as well as to the B5-positive peri-nuclear compartment. Moreover, we found that loss of E2 resulted in a large reduction in the spread of the virus towards the cell periphery, as the protein is required for normal IEV morphogenesis. Our observations in ΔE2L-infected cells are in agreement with recent observations of Domi *et al*. (2008) who have shown that deletion of the E2 gene from the virus genome results in a dramatic reduction in the formation of extracellular virus particles. This reduction severely impairs the cell-to-cell spread of infection and helps to explain the small plaque phenotype of the ΔE2L virus on confluent cell monolayers. Moreover, Domi *et al*. (2008) found that loss of E2 also resulted in defects in IEV morphogenesis, including the presence of aberrantly wrapped virus particles. Our combined observations suggest that E2 plays an important but undefined role in IEV particle assembly.

Our observation that E2 and F12 require each other for their recruitment to IEV particles suggests that they are working as a complex. This would explain why the ΔE2L and ΔF12L viruses have a similar phenotype, including identical defects in IEV morphogenesis in HeLa cells. This is in direct contrast to the previous analysis of ΔF12L-infected BS-C-1 cells in the electron microscope, which did not reveal any obvious defects in IEV morphogenesis (van Eijl *et al*., 2002). These differences cannot be due to cell type as we obtained similar results in both HeLa and BS-C-1 cells infected with ΔF12L (Figs S2 and S3). One possible explanation may reside in the thickness of the sections. As pointed out by Domi *et al*. (2008) it is difficult to assess the completeness of wrapping in thin sections in the electron microscope. Because of the chromatic error correction system of our electron microscope, we are able to obtain sharp high-contrast images on specimens that are 120 nm rather than the more usual 80 nm. This 50% increase in specimen thickness will undoubtedly enhance the ability to assess IEV morphogenesis, as the wrapping membranes and their connections are more visible. Consistent with this, detailed examination of fig. 8c of van Eijl *et al*. (2002) reveals suggestions of membrane connections similar to the ones we have observed in ΔF12L infected cells.

Our observations indicate that E2 and F12 function as a complex during vaccinia infection. Unfortunately, sequence analysis and secondary structural predictions of E2 or F12 do not reveal any obvious motifs or domains that might be responsible for complex formation or their recruitment to the B5 peri-nuclear membrane compartment or IEV. Furthermore, neither protein is observed on the trans-Golgi when coexpressed in non-infected cells (data not shown), suggesting that their localization in infected cells is likely to be dependent on the presence of other viral protein(s). Consistent with this hypothesis, very recent observations using yeast two-hybrid analysis and GST pull-down assays have demonstrated that residues 351–458 of F12, but not the full-length protein, can interact directly, although weakly, with A36 (Johnston and Ward, 2008). Moreover, this interaction, which involves residues 91–111 of A36, overlaps exactly with the binding site of A33 and kinesin-1 light chain on A36 (Ward *et al*., 2003; Ward and Moss, 2004; Johnston and Ward, 2008). Curiously, deletion of residues 351–458 of F12 has the same effect as loss of the complete protein, including reduction in extracellular virus production, as well as an absence of actin tails and B5R staining on the plasma membrane, all of which are consistent with the very small plaque phenotype of both these viruses (Zhang *et al*., 2000; Johnston and Ward, 2008).

During our study we found that IEV morphogenesis is severely impaired in ΔF12L- as well as ΔE2L-infected cells. In contrast, IEV morphogenesis is indistinguishable in the ΔA36R-and WR-infected cells (Parkinson and Smith, 1994; Röttger *et al*., 1999; Smith *et al*., 2002). If A36 is essential for F12 recruitment, and presumably E2, then IEV morphogenesis should also be compromised in ΔA36R-infected cells. How can we explain this apparent discrepancy? The simplest explanation is that the E2–F12 complex has additional interactions besides F12 binding A36. The most likely candidates for binding partners are proteins of the IEV, which are known to form a variety of complexes with each other, through a number of different interactions (Röttger *et al*., 1999; Wolffe *et al*., 2001; Smith *et al*., 2002; Perdiguero and Blasco, 2006; Perdiguero *et al*., 2008). Interestingly, the wrapping defects we have observed in the absence of E2 and F12 are somewhat reminiscent to that seen when the gene encoding the IEV protein, A33, is deleted from the viral genome (Roper *et al*., 1998). If the E2–F12 complex binds IEV proteins that themselves have multiple and cooperative interactions, then why does deletion of residues 351–458 of F12 have such a large impact? Secondary structure predictions suggest that F12 adopts a globular structure that is rich in alpha helices. In the absence of a structure it is impossible to rule out that deletion of residues 351–458, which is predicted to be largely alpha helical in nature, does not also disrupt its interaction with E2 and/or other IEV proteins as a result of loss of the structural integrity of the protein. Such disruption would explain why deletion of residues 351–458 leads to a phenotype that is essentially identical to loss of the complete protein (Johnston and Ward, 2008).

It is clear from our observations that an E2–F12 complex plays an important role in IEV morphogenesis. The accumulation of partially wrapped IEV and dense tubular membrane network in the peri-nuclear region in the absence of E2 or F12 would suggest that both proteins may have important interactions with host machinery involved in the regulation of membrane traffic during infection. The challenge is now to unravel their role in this critical phase of the virus life cycle.

## Experimental procedures

### Recombinant viruses

#### GFP-F12

A region corresponding to 1084 base pairs (bp) upstream of the F12L gene was amplified from WR genomic DNA by PCR and placed upstream of GFP-F12L in pBS SKII. A recombinant virus was isolated by rescue of ΔF12L (Zhang *et al*., 2000) and selecting for a recovery in plaque size similar to WR as well as expression of GFP-F12 during five rounds of plaque purification.

#### GST-3C2-FLAG-F12

A region corresponding to 1060 bp upstream of the F12L gene was amplified from WR genomic DNA and placed upstream of GST in a GST-3C2-FLAG-F12L in pBS SKII. A recombinant virus was derived by rescue of ΔF12L, and selecting for a recovery in plaque size similar to that of WR during five rounds of plaque purification.

#### *Δ*E2L

A region corresponding to 311 bp upstream of the E2L gene was amplified by PCR from WR genomic DNA. Sequence corresponding to the final 308 bp of E2L was also amplified. These products were then cloned into pBS SKII such that they flanked a cassette consisting of gpt and Cherry proteins under the control of a vaccinia synthetic early/late promoter pE/L (Chakrabarti *et al*., 1997). The final 308 bp of E2L gene was included in the construct to ensure that the promoter of the E1L, which is contained within E2L, would not be disrupted. A virus lacking E2L was derived by transient transfection of the construct into a WR background, followed by five rounds of plaque purification using mycophenolic acid selection and red fluorescence to identify plaques induced by the ΔE2L virus.

#### GFP-E2L viruses

A region corresponding to 311 bp upstream of the E2L gene initiator codon was amplified from WR genomic DNA and placed upstream of GFP in a GFP-E2L in pBS SKII. A recombinant virus expressing GFP-E2L was then derived by transfection of the targeting construct into a WR background, selecting for expression of GFP during five rounds of plaque purifications selecting for GFP fluorescence. The same process was using to introduce GFP-E2L into the ΔF12L virus.

#### B5R-YFP viruses

A region corresponding to B5R and the 300 bp upstream of the ORF was amplified by PCR from WR genomic DNA and fused to YFP. A region corresponding to 300 bp downstream of B5R stop codon was amplified by PCR cloned downstream of YFP in a B5R-YFP expression construct. To produce WR-B5R-YFP, a ΔB5R virus (Engelstad *et al*., 1992) was rescued, selecting for increase in plaque size to that of WR and expression of YFP. To produce ΔF12L-B5R-YFP, the B5R-YFP cassette was introduced into the ΔF12L background.

The fidelity of all viruses was verified by sequencing and Western blot. In the case of ΔE2L and GFP-E2 viruses Western blots were performed on glutathione pull of extracts from infected cells expressing GST-F12 to enrich for the protein, as E2 is expressed at low levels.

### Infections, transfections and immunofluorescence analysis

HeLa cells plated on fibronectin-coated coverslips or dishes were infected with the indicated viruses as previously described (Arakawa *et al*., 2007a). When required, a plasmid expressing RFP-A3 or GFP-F12 under control of the synthetic early/late promoter (Chakrabarti *et al*., 1997; Frischknecht *et al*., 1999) was transfected 1–2 h later. Cells were processed for immunofluorescence and Western analysis as described previously (Arakawa *et al*., 2007b). Images were prepared for publication using the Adobe photoshop and illustrator packages (Adobe, CA, USA).

### Antibodies

Monoclonals C3 and 19C2 were used in immunofluorescence and Western blot analysis to detect A27 and B5 viral proteins respectively (Hiller and Weber, 1985; Rodriguez *et al*., 1985). A33, A36 and F13 polyclonal antibodies have been described previously (Röttger *et al*., 1999; Rietdorf *et al*., 2001). Anti-F12 antibodies were produced in rabbits by immunization with a peptide corresponding to amino acids residues 614–634 of F12 (KELYVSSSYKDINESMSQMVK) coupled via an additional N-terminal Cys-Gly-Gly linker to Keyhole Limpet hemocyanin using the Imject activated immunogen conjugation kit (Pierce Chemical). F12 antibodies were affinity-purified on the same F12 peptide, which was coupled via the free cysteine to a SulfoLink column (Pierce Chemical). The specificity of the resulting F12 antibodies was confirmed by Western analysis on HeLa cells infected with WR and ΔF12L virus. Anti-E2 antibodies were produced as above using a peptide corresponding to amino acids 722–737 of E2 (CVETILDNNQSFKSSK).

### Interactions between F12 and E2

To identify F12 interacting partners, four 15 cm dishes of 293T cells were infected with either WR or WR-GST-3C-FLAG-F12L viruses. Sixteen hours post infection, cells were washed twice with PBS and lysed in 50 mM Tris pH 7.5, 150 mM NaCl, 1% Triton-X 100 and a protease inhibitor cocktail. Lysates were clarified by being centrifuged at 100 000 *g* for 30 min before being twice passed through a column containing glutathione resin. Columns were washed with 50 mM Tris pH 7.5 and 150 mM NaCl until no protein could be detected in the flow though in Bradford assay. Bound material was eluted from the resin using glutathione, concentrated by precipitation with TCA and resuspended in SDS loading buffer. Gel was stained using SilverQuest (Invitrogen) and bands unique to WR-GST-3C-FLAG-F12 were excised and analysed by the Taplin Biological Mass Spectrometery Facilty (Harvard University, Boston, USA).

Pull-downs from infected cells were performed on extracts derived from single 10 cm dishes of 293T cells infected with WR and transfected with the indicated combinations of constructs expressing GST, GST-F12, GST-E2, GFP, GFP-E2 and GFP-F12 under control of the pE/L promoter. Cells were lysed and GST-tagged proteins bound to glutathione-coated beads, as described above. Beads were washed three times with lysis buffer (in the absence of detergent), boiled in SDS-loading buffer and analysed by Western blot using antibodies against GST, GFP, E2 and F12.

GST, GST-E2 and His-F12 were produced in bacteria using the pMW T7 vector as previously described (Boëda *et al*., 2007). GST- and GST-E2-coated beads were then added to the soluble bacterial extract containing His-F12. Beads were washed and bound material was analysed by Western blot as described previously (Boëda *et al*., 2007).

### Electron microscopy

Cells were grown on 13 mm fibronectin-coated glass coverslips and fixed in 1.5% glutaraldehyde/2% paraformaldehyde in 0.1 M phospate buffer, pH 7.4 for 30 min at room temperature. Cells were then post-fixed in 1.5% potassium ferrocyanide/1% osmium for 1 h and stained with 1% tannic acid in 0.05 M sodium cacodylate pH 7.4 for 45 min. Coverslips were dehydrated stepwise through ethanol, infiltrated with 50:50 propylene oxide : epon followed by two changes of pure resin and embedded ‘en face’ at 60°C overnight. Sections of ∼120 nm were collected using an UCT ultramicrotome (Leica Microsystems UK), post-stained with lead citrate and viewed using a Tecnai G2 Spirit 120 kV transmission electron microscope (FEI Company) and imaged with an SC1000 Orius CCD camera (Gatan UK).

### Quantitative analysis of actin tail formation and plaque sizes

Plaque areas were measured at 48 h post infection by immunofluorescence as the area covered B5-positive cells (*n* = 20) or by crystal violet staining at 96 h as described previously (Arakawa *et al*., 2007b). Number of actin tails per cell was determined in 150 infected cells as described previously (Weisswange *et al*., 2009).

### Fluorescence microscopy and image analysis

Images from live cells grown in 35 mm glass bottom MatTek dishes and infected for 8 h were collected using a Cascade II 512B cooled CCD camera (Photometrics, AZ, USA) on an Axiovert 200 microscope using an Apochromat 63/1.40 NA Oil lens (Carl Zeiss, Germany) under the control of Metamorph (Molecular Devices). All signals from GFP- or YFP and RFP-tagged proteins were acquired simultaneously using a Dual View filter system (Optical Insights, NM, USA) at two frames per second. Image sequences and rates of virus movement were determined using Metamorph and supplementary movies assembled and annotated using Adobe After Effects (Adobe, CA, USA).

## Supporting Information

**Fig. S1.** GFP-F12 is associated with all IEV. Immunofluorescence images of WR-GFP-F12-infected HeLa cells showing that GFP-F12 is associated with all intracellular F13-positive virus particles. Pink arrows indicate IEV that are F13-, DAPI- and GFP-F12-positive but negative for extracellular B5. White arrows indicate CEV that are positive for F13, DAPI and extracellular B5 but negative for GFP-F12. Yellow arrow points to a F13-positive vesicle that lacks signals from DAPI, GFP-F12 and extracellular B5. Orange arrow shows IMV (DAPI-positive only). The scale bar is 5 μm.

**Fig. S2.** E2 and F12 are required for IEV morphogenesis. Electron micrographs showing the peri-nuclear wrapping compartment in ΔE2L- and ΔF12L-infected HeLa cells at 8 h post infection. Red arrows show examples of partially wrapped IMV. Scale bars = 0.1 μm top left panel and 0.5 μm all other panels.

**Fig. S3.** E2 and F12 are required for IEV morphogenesis in BSC-1 Cells. Electron micrographs showing the peri-nuclear wrapping compartment in ΔE2L- and ΔF12L-infected BSC-1 cells at 10 h post infection. Red arrows highlight examples of partially wrapped IMV. Scale bars = 0.5 μm.

**Movie S1.** GFP-F12 is associated with IEV undergoing rapid linear movement towards the cell periphery. Left panel = GFPF12, middle = RFP-A3, right = Merge. Yellow arrow highlights an IEV (positive for GFP-F12 and RFP-A3) moving from the site of wrapping towards the cell periphery. Red arrow shows a virus (presumably IMV) moving in the absence of F12. Time in seconds and scale bar are indicated in the left panel.

**Movie S2.** The movie shows the loss of GFP-F12 during the transition from microtubule to actin-based motility. Left panel = GFP-F12, middle = RFP-A3, right = Merge. Yellow arrow highlights an IEV (positive for GFP-F12 and RFP-A3) moving towards the cell periphery. The arrow changes from yellow to red as F12 vanishes and the virus switches to a slower more processive movement. Time in seconds and scale bar are indicated in the left panel.

**Movie S3.** The arrowhead indicates an IEV (B5-YFP- and RFPA3- positive) undergoing rapid linear movements in the absence of F12. Left panel = B5-YFP, middle = RFP-A3, right = Merge. Time in seconds and scale bar are indicated.

**Movie S4.** The movie shows the loss of GFP-E2 during the transition from microtubule to actin-based motility. Left panel = GFP-E2, middle = RFP-A3, right = Merge. Yellow arrow highlights an IEV (positive for GFP-E2 and RFP-A3) moving towards the cell periphery. The arrow changes from yellow to red as E2 vanishes and the virus switches to a slower more processive movement. Time in seconds and scale bar are indicated in the left panel.
