# Supplementary figures and images for: An E2–F12 complex is required for intracellular enveloped virus morphogenesis during vaccinia infection
Source: Cell Microbiol. 2009 Feb 27;11(5):808–24. doi: 10.1111/j.1462-5822.2009.01296.x (PMC2688674; doi:10.1111/j.1462-5822.2009.01296.x)

**Ex-B5** **GFP-F12** **F13**

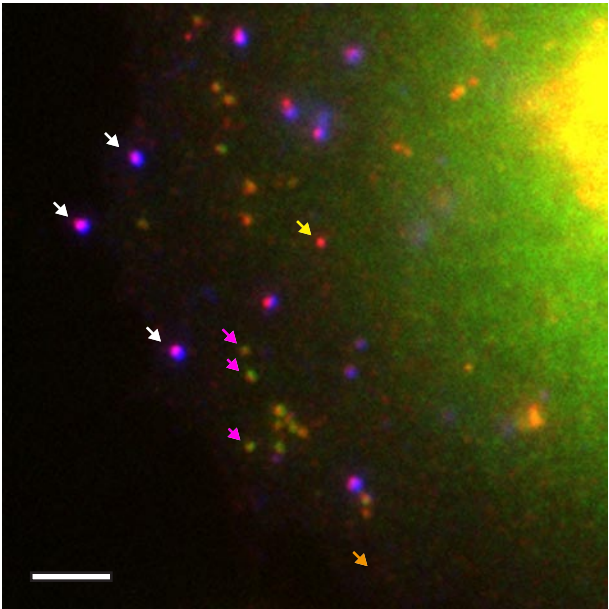

**GFP-F12** **F13**

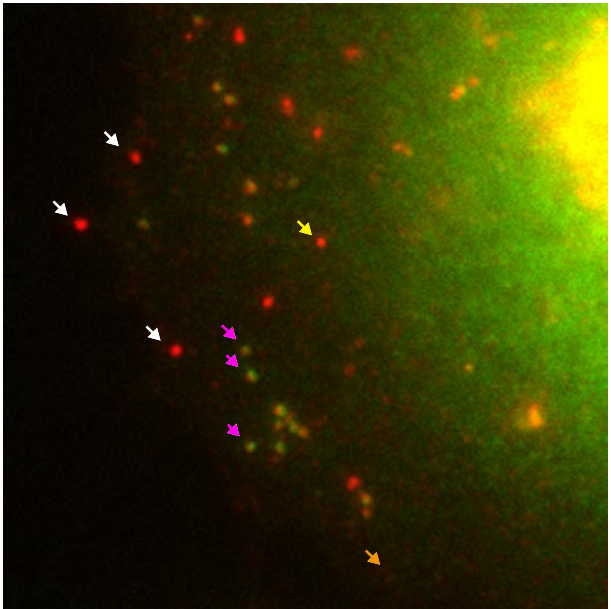

**GFP-F12**

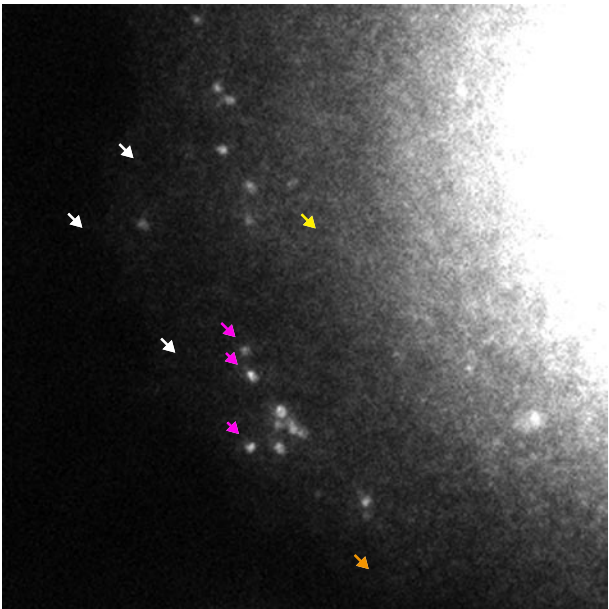

**F13**

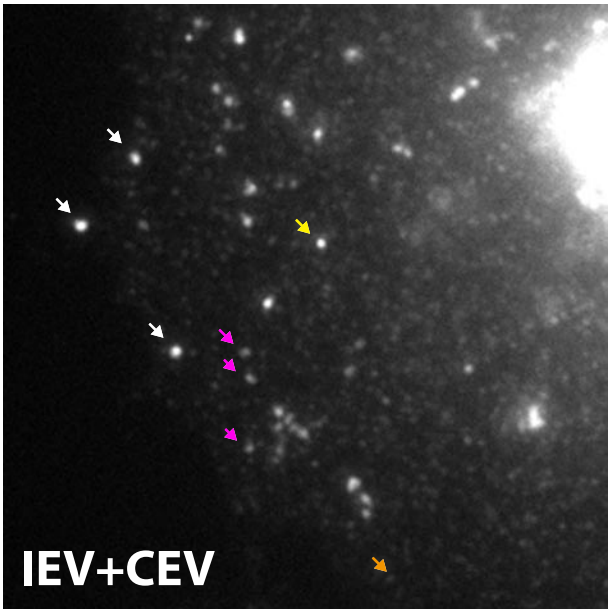

**IEV+CEV**

**DAPI**

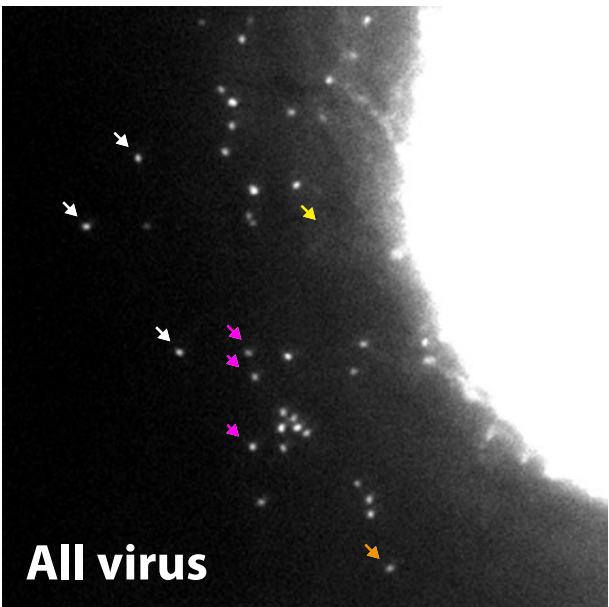

**All virus**

**Extracellular B5**

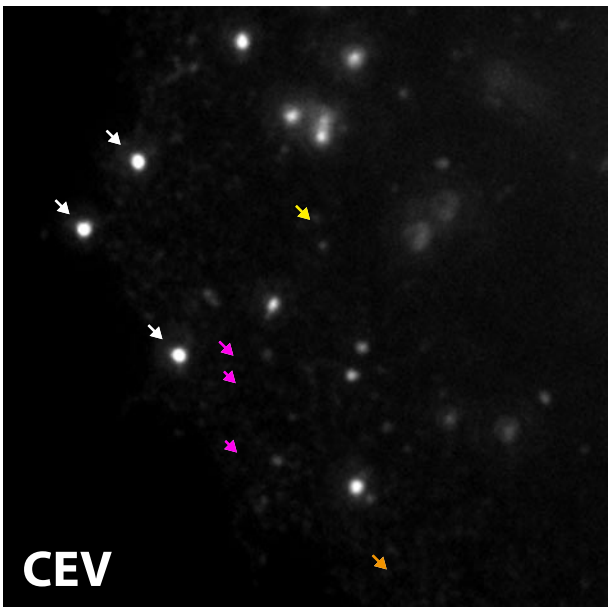

**CEV**

Supplement: Supplementary file 1 [file cmi0011-0808-SD1.pdf]

HeLa

$\Delta E2L$

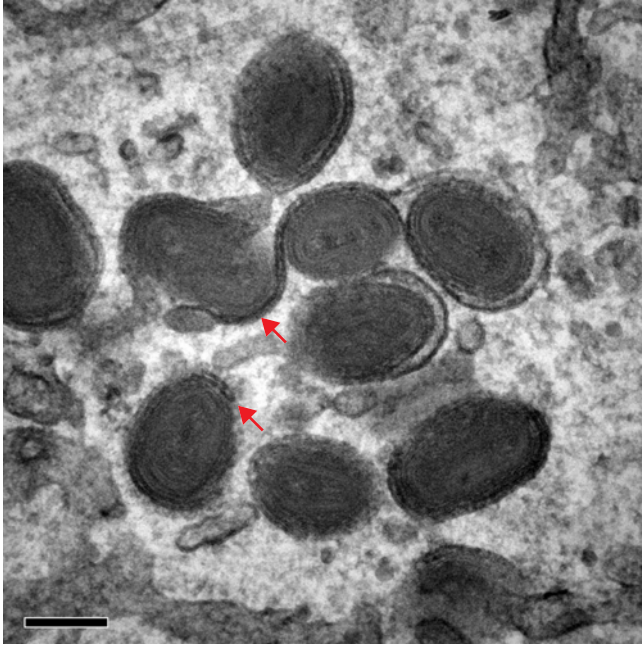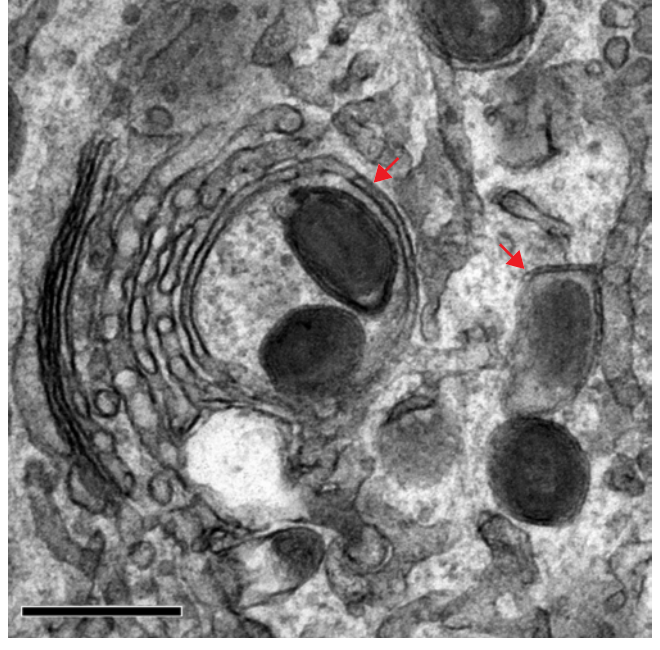

$\Delta F12L$

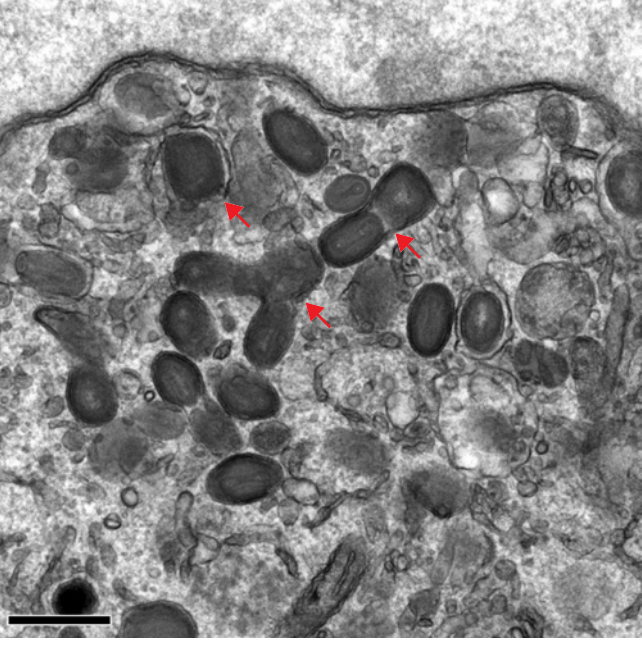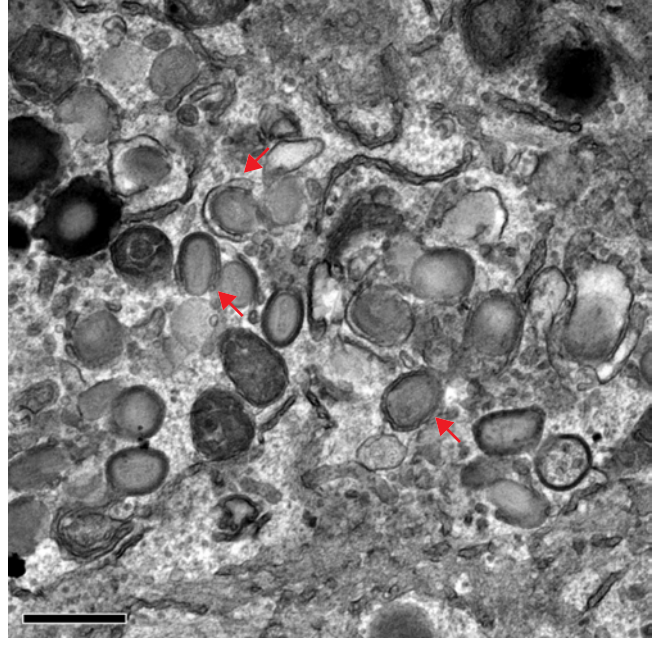

$\Delta F12L$

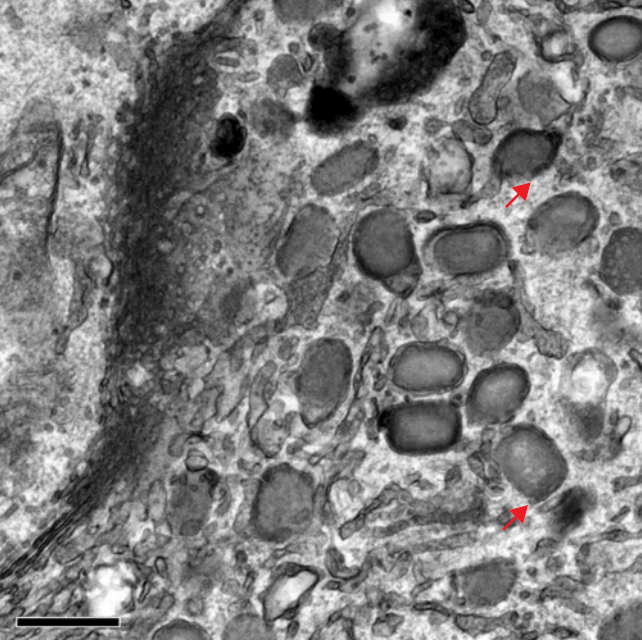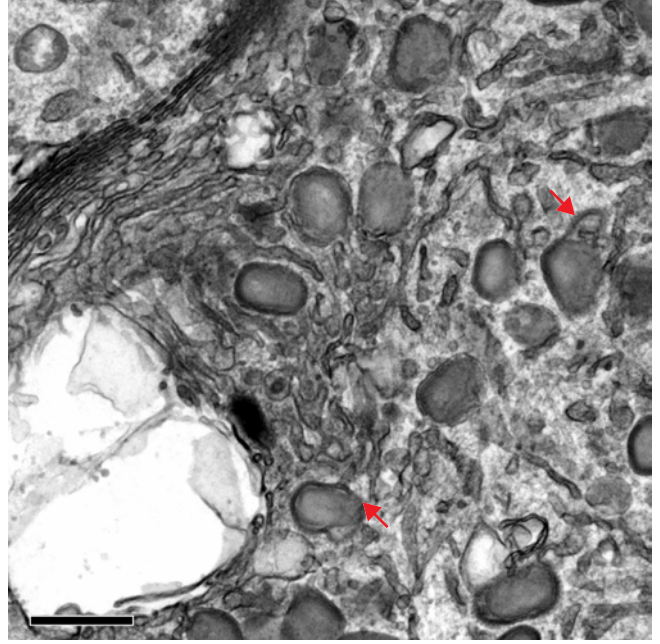

Supplement: Supplementary file 2 [file cmi0011-0808-SD2.pdf]

BSC-1

$\Delta E2L$

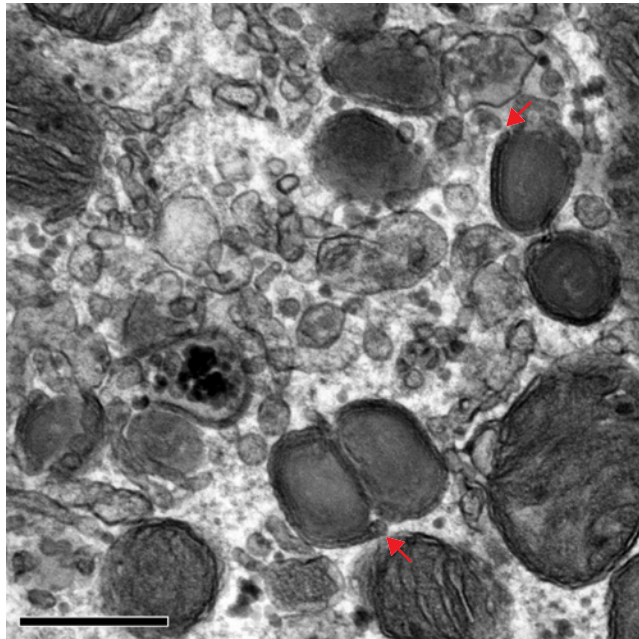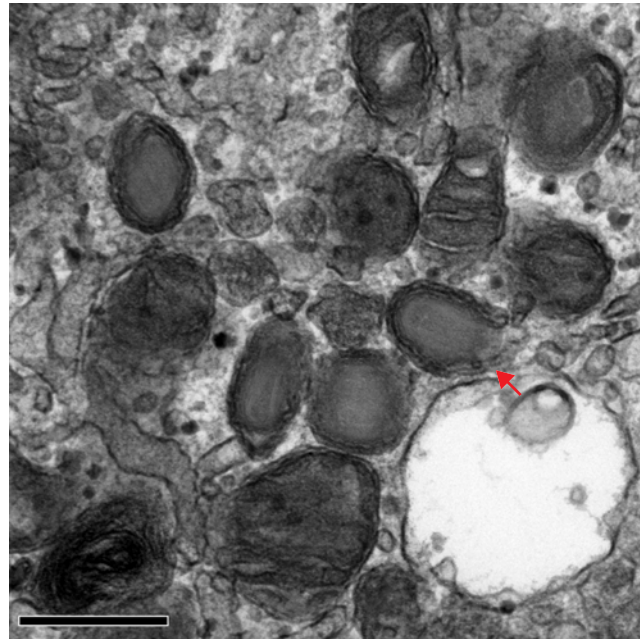

$\Delta F12L$

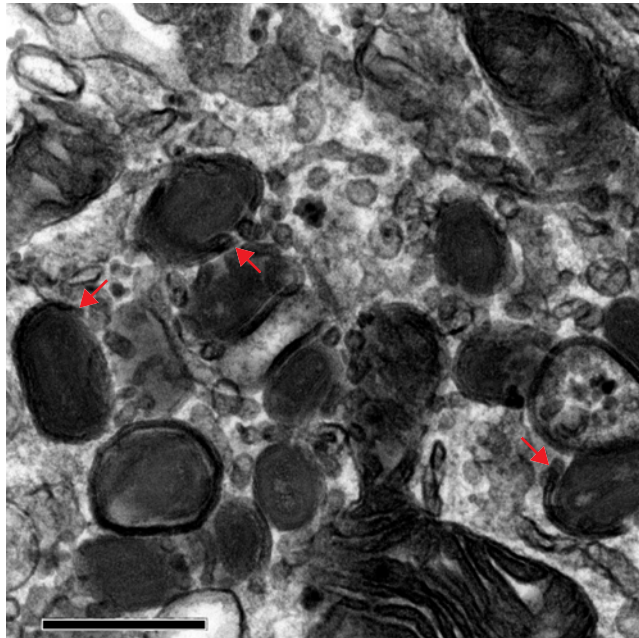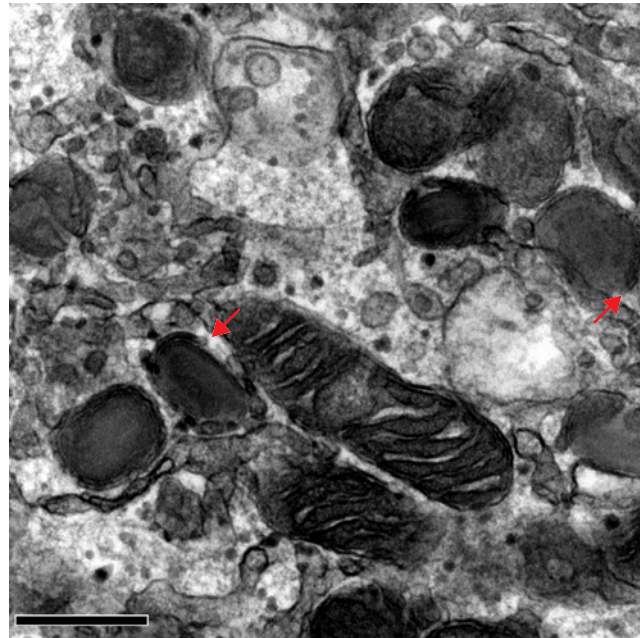

Supplement: Supplementary file 3 [file cmi0011-0808-SD3.pdf]
